# Spread of anti-malarial drug resistance: Mathematical model with implications for ACT drug policies

**DOI:** 10.1186/1475-2875-7-229

**Published:** 2008-11-02

**Authors:** Wirichada Pongtavornpinyo, Shunmay Yeung, Ian M Hastings, Arjen M Dondorp, Nicholas PJ Day, Nicholas J White

**Affiliations:** 1 Mahidol – Oxford Tropical Medicine Research Unit, Faculty of Tropical Medicine, Mahidol University, Bangkok, Thailand; 2 Health Policy Unit, London School of Hygiene and Tropical Medicine, Keppel Street, London, UK; 3 Liverpool School of Tropical Medicine, Pembroke Place, Liverpool, UK; 4 Centre of Clinical Vaccinology and Tropical Medicine, Nuffield Department of Clinical Medicine, University of Oxford, Oxford, UK

## Abstract

**Background:**

Most malaria-endemic countries are implementing a change in anti-malarial drug policy to artemisinin-based combination therapy (ACT). The impact of different drug choices and implementation strategies is uncertain. Data from many epidemiological studies in different levels of malaria endemicity and in areas with the highest prevalence of drug resistance like borders of Thailand are certainly valuable. Formulating an appropriate dynamic data-driven model is a powerful predictive tool for exploring the impact of these strategies quantitatively.

**Methods:**

A comprehensive model was constructed incorporating important epidemiological and biological factors of human, mosquito, parasite and treatment. The iterative process of developing the model, identifying data needed, and parameterization has been taken to strongly link the model to the empirical evidence. The model provides quantitative measures of outcomes, such as malaria prevalence/incidence and treatment failure, and illustrates the spread of resistance in low and high transmission settings. The model was used to evaluate different anti-malarial policy options focusing on ACT deployment.

**Results:**

The model predicts robustly that in low transmission settings drug resistance spreads faster than in high transmission settings, and treatment failure is the main force driving the spread of drug resistance. In low transmission settings, ACT slows the spread of drug resistance to a partner drug, especially at high coverage rates. This effect decreases exponentially with increasing delay in deploying the ACT and decreasing rates of coverage. In the high transmission settings, however, drug resistance is driven by the proportion of the human population with a residual drug level, which gives resistant parasites some survival advantage. The spread of drug resistance could be slowed down by controlling presumptive drug use and avoiding the use of combination therapies containing drugs with mismatched half-lives, together with reducing malaria transmission through vector control measures.

**Conclusion:**

This paper has demonstrated the use of a comprehensive mathematical model to describe malaria transmission and the spread of drug resistance. The model is strongly linked to the empirical evidence obtained from extensive data available from various sources. This model can be a useful tool to inform the design of treatment policies, particularly at a time when ACT has been endorsed by WHO as first-line treatment for falciparum malaria worldwide.

## Background

For the past half-century, the malaria parasites of humans have been under tremendous selection pressure to evolve mechanisms of resistance to the prevailing anti-malarial drugs. Chloroquine and, increasingly, sulphadoxine-pyrimethamine (SP), have become largely ineffective as monotherapy for the treatment of *Plasmodium falciparum *malaria in much of the world. The World Health Organization (WHO) now recommends artemisinin-based combination therapy (ACT) as first-line treatment for all falciparum malaria in endemic areas [1].

The artemisinin-based combinations are efficacious, rapidly acting, well-tolerated and safe. They are available in various formulations, which are generally given over three days. The various forms of ACT are effective against both asexual and early sexual parasite stages [2], and, thereby, reduce transmissibility [3,4]. The contribution of reduced transmissibility of individual treated infections to overall transmission depends on the proportion of transmissible infections that are treated and the degree of 'saturation' in the transmission dynamics. So far, substantial *in-vivo *resistance to artemisinin derivatives has not yet been confirmed, and stable resistance has been very difficult to produce in the laboratory [5]. As for any combination therapy, which involves two effective drugs from different classes, both component drugs protect each other from the development of drug resistance, whilst present at effective concentrations. This should prolong their useful lifespan provided that the individual components are not widely available as monotherapies [6].

Although malaria-endemic countries are switching to ACT with increasing momentum, even at prices as low as US$1 per dose, it is still too costly for communities and governments in poorer countries (5 – 10 times higher than the prices of chloroquine or SP in Africa [7]). Doubts have been raised about the actual operational effectiveness of ACT when implemented in "real-life" situations, where infrastructures are weak, access to health care is poor, and there is widespread inappropriate use of anti-malarial drugs [8]. Although providing easy access to very low cost ACT in the private sector or free ACT in the public sector may achieve this aim, it has to be balanced against the costs and risks of the widespread use of such combinations. In particular, if artemisinins are used on their own and not in co-formulation with an effective partner drug, then there is a much greater risk of drug resistance arising to this precious class of drugs. Questions remain about the choice of combination therapy and timing of policy change. Finally, an important additional benefit of ACT in low-transmission areas is the potential ability to reduce malaria transmission and thus the incidence of malaria. Enthusiasm for the deployment of ACT in high transmission settings is tempered by the expectation that this deployment is less likely to translate into reduced malaria incidence in these settings.

In the current study, a comprehensive mathematical model describing malaria transmission and evolution of anti-malarial drug resistance is constructed to answer the questions on treatment strategies focusing on the deployment of ACT in different transmission settings.

## Methods

### Modeling transmission dynamics and the spread of anti-malarial drug resistance

The development of drug resistance is a two-step process, the *de novo *or the initial emergence of resistance, and its subsequent spread. Resistance spreads because of the higher reproductive rate of resistance infections in the presence of anti-malarial drugs. In this paper, the focus is on modeling the spread of resistance assuming that drug resistance has already emerged among the human population. Combinations prevent resistance by preventing *de novo *emergence. Modeling the *de novo *emergence of drug resistance is discussed elsewhere [9,10].

Transmission and anti-malarial drug resistance are modeled on an age-structured population-based model, where the size and age-structure of the human population were assumed to be constant over time and based on an average African age-structure . A stochastic component of new imported infections (migrant) is added to the model making it more realistic.

The dynamic of mosquito population was summarized into vectorial capacities (VC) and estimated from several epidemiological studies. It is assumed that all humans are equally attractive to biting mosquitoes. Malaria is transmitted when a susceptible human is bitten by an infected Anopheline mosquito. The rate at which a susceptible person becomes infected or the inoculation rate is a function of contact rate with infective mosquitoes and level of host susceptibility, based on the formula given by Dietz [11] (see equation 1, Additional File 1). By assuming that all mosquito biting vectors are equally susceptible, human infectiousness to mosquitoes is determined solely by the gametocyte density in humans with a non-linear relationship shown from the malariatherapy study by Jeffery and Eyles [12] (see equation 5, Additional File 1). Human infectiousness can be estimated as the model handles malaria like a "macro" parasite by quantifying the density of infection in the human host. The *in-vivo *effect of drugs on parasite density can be measured, allowing quantification of the pharmacodynamic properties of anti-malarial drugs [13]. The average gametocytaemia depends directly on the average parasite biomass by age group and the estimated gametocyte switch rate (i.e. probability that an asexual parasite develops to a sexual parasite). For simplicity, the gametocyte switching rate (GSR) was assumed to be uniform among asexual parasites and infections, but vary among anti-malarial drugs, and between primary and recrudescent infections.

The dynamic in human mainly consists of the development of immunity by age and transmission intensity. Making the model data-driven, the parameters for immunity functions and malaria infection in the human host (asymptomatic, symptomatic and recrudescent infections) were obtained from clinical, laboratory and epidemiological studies. Appropriate curve fitting including multiple linear and non-linear regressions was fitted to different data from age-stratified epidemiological studies in areas with different transmission intensities to derive immunity functions of age and EIR (Additional File 2). Stepwise selection using the Akaike Information Criterion (AIC) was used to identify the best fit in the case of non-nested functions and the maximum-likelihood ratio test was used for nested functions. These relationships represent the acquisition of immunity to malaria by age and frequency of malaria exposures. Different facets of malaria immunity are incorporated into this model i.e. reducing host susceptibility to infection [14,15], reducing the level of (largely asymptomatic) parasitaemia in infected people [16,17], reducing the likelihood of fever and other symptoms in infected patients [18-20], reducing the treatment failure rate for a particular level of anti-malarial drug resistance [21-26], and increasing the recovery rate of an established infection i.e. shortening the duration of infection [27-29] (Table S7, Additional File 2). Some immunity functions were not measured directly from epidemiological studies (i.e. host susceptibility, duration of infections and treatment failure), so a normalized function of age-stratified parasite density was used to estimate the relationship between age and host susceptibility and between age and duration of infections by specifying initially the "maximum host susceptibility" in a non-immune person and the "maximum duration" of *treated *and *untreated *infections. The host susceptibility and the duration of infection for any given age group in any transmission intensity setting are then determined by the shape of the immunity curve. As the shape of the age effect on treatment failure is similar to the relationship between age and severe malaria [21], the same technique to the normalized function of age-stratified risk of severe malaria is applied to adjust the treatment failure rates for any given age group in any transmission intensity setting. The maximum value of duration of infection and the maximum value of treatment failure are dependent on treatment type, drug resistance, and likelihood of patient adherence to therapy. Adherence is incorporated in the model by adjusting down the expected failure rates of treated infections. Multiple recrudescences are treated as one continuous recrudescence, and overall infectiousness is calculated from the area under the gametocyte-time curve (AUCgam). Multiple infections in an individual or "superinfection" is not considered in this model.

The population with selective residual anti-malarial drug concentrations focused on this model is the proportion with concentrations in the blood which prevent establishment of new drug sensitive infections but allow establishment of resistant infections. This proportion of population was based on published literature (equation 6 – 7, Additional File 1).

### Model simulations and outputs

**Figure 1:**
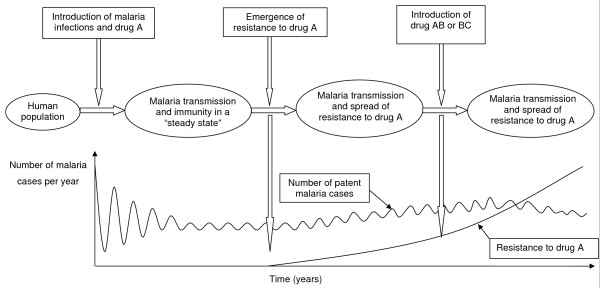
**Schematic diagram of the biological model through time**. The model progresses from steady state through to the introduction of resistance and changes in drug policy.

At the outset, a human population is assumed to have little or no exposure to malaria and, therefore, the population has no immunity to malaria. Infected mosquitoes bite randomly and infect humans with drug susceptible infections i.e. malaria infections initially are all drug-sensitive and symptomatic humans receive only monotherapy (drug A). As the population becomes exposed to malaria and gains some level of immunity, the model updates the age-stratified immunity of the population according to the Entomological Inoculation Rate (EIR) (which varies with VC and the infective human population (see equation 8, Additional File 1)), and is allowed to run until a steady state is reached (Figure 1). A steady state point is defined as the point at which the number of new malaria cases (i.e. excluding imported cases) has varied day to day by less than 1% over a year. Any changes in malaria prevalence and levels of drug resistance thereafter can reasonably be assumed to result from introduction of drug resistance and the impact of the different treatment strategies. Once the steady state is obtained, the resistance to drug A is introduced, either as importation of a small number of resistant infections or by the *de novo *emergence of resistance based on available clinical and laboratory data. At a specified threshold of resistance to drug A (defined as a model parameter) artemisinin or its derivatives, or a completely new drug can also be introduced and used in a combination with drug A, or it can be used as a monotherapy. Resistance can then be tracked for a specified length of 10 years after steady state to gauge the impact on model outcomes over time. The model provides a number of outputs including estimated EIR, proportion of symptomatic infections, proportion of treatment failure, malaria prevalence and percentage of resistant infections. Those relevant to policy, the proportion of infections with resistant parasites, the malaria prevalence, and the incidence of malaria are presented here. The sensitivity of the model was tested in the four baseline scenarios (scenario A – D). The details of fixed and variable parameters with their respective distributions are given in Tables S1 – S6, Additional File 2. Each scenario is repeatedly run for 5,000 simulations with a unique set of parameters selected using the Latin Hypercube Sampling technique (LHS). The Coefficients of Variation (CVs), which determine the uncertainties of the model outcomes (Table S9, Additional File 3), and the Partial Rank Correlation Coefficients (PRCCs), which identify influential factors, were calculated (Table S10, Additional File 3). Full technical details can be found in [30].

## Results

### Resistance, transmission intensity and ACT coverage

Model consistency and model sensitivity are explored in four baseline scenarios (low transmission setting with low ACT coverage (scenario A), low transmission setting with high ACT coverage (scenario B), high transmission setting with low ACT coverage (scenario C) and high transmission with high ACT coverage (scenario D)). The model consistency is expressed as the Coefficient of Variation (CV) i.e. the variations in the model outputs given that there are uncertainties in the input parameter estimated (Tables S9, Additional File 3). For sensitivity, the Partial Rank Correlation Coefficient (PRCC) was used to describe the correlation between the model output to each input parameter (Tables S10, Additional File 3). ACT coverage is defined as the proportion of ACT treatments among all symptomatic treated infections.

In the low transmission setting (EIR < 1), with low ACT coverage, variations in the estimated prevalence of malaria stay consistently high over time (CV ~ 90%) after the steady state is obtained, while the variation in the estimated resistance falls substantially till year 8 (CV ~ 9%) with resistance approaching fixation at 100% in our 10-year time horizon. In low transmission settings with high ACT coverage, the mean prevalence of malaria stays below 1% over 10 years. Migration plays an important role in sustaining malaria. Without imported cases, malaria is readily eradicated. Mean resistance increases at a slower rate than in a low coverage setting, reaching 80% in year 10. Compared to scenario A, variation in the estimated prevalence is slightly lower (CV ~ 75%) while variation in the rate of resistance is higher (CV > 30%). This indicates some levels of uncertainty in the consequences of deploying a high coverage of ACT on the malaria prevalence and the rate of resistance. Once resistance to the slowly eliminated partner drug has emerged the spread of drug resistance and the malaria prevalence could be slowed down only by deploying ACT at a high coverage rate while the resistance prevalence is still reasonably low.

In the low transmission setting, VC was the most influential parameter affecting malaria prevalence (PRCC ~ 0.6), while VC (PRCC ~ 0.7) and the proportion of treated infections (PRCC ~ 0.4) were the most influential parameters affecting the spread of drug resistance.

In the high transmission setting (EIR > 100), malaria cannot be eradicated by anti-malarial treatment of symptomatic cases alone because the major transmission reservoir is in asymptomatic persons who do not take anti-malarial drugs. In the high transmission setting with low ACT coverage, malaria prevalence increases from 36% to 44% within 10 years due to the spread of resistance. Resistance spreads more slowly compared to the low transmission setting, reaching 80% in year 10. Variation in estimated prevalence is small (CV < 30%) while variation in the rate of resistance is consistently high (CV > 45%) compared to estimates in the low transmission setting. Both outcomes and their variations are unaltered by deploying a high coverage of ACT. Treatment of symptomatic infections in the high transmission setting has much less effect than in the low transmission setting. Consequently, the spread of drug resistance is driven by the fraction of the population with some residual anti-malarial drug in their blood and not by treatment failure.

In the high transmission setting, the parameters influencing malaria prevalence are the characteristics of the infection in immune subjects with untreated infections. These are the parasite biomass (PRCC ~ 0.4), the gametocyte switch rate (PRCC ~ 0.4) and the duration of infection (PRCC ~ 0.3). The proportion of residual drug in the population (PRCC ~ 0.9) is the dominant factor driving the spread of drug resistance in this setting. The strong correlation between the fraction of population with residual drug concentrations and the levels of resistance from this model suggests that controlling the use of presumptive treatment and encouraging the use of combination therapy with matching half-lives to reduce the selective window would slow the spread of resistance down within this setting.

### Scenario A: Effects on resistance of delaying the policy change the ACTs

**Figure 2:**
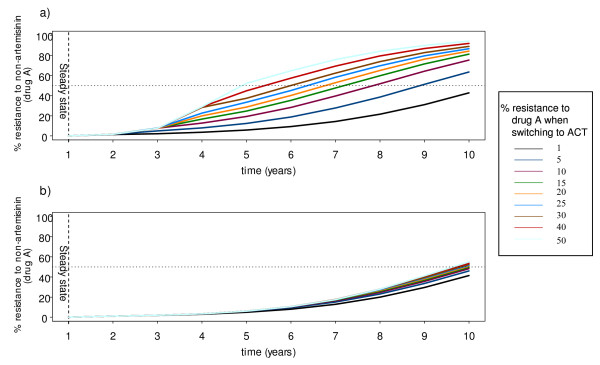
**The model-predicted spread of SP resistance**. The predicted spread of resistance to mefloquine when combined with artesunate at different level of resistance. Figure 2A shows the result in low transmission setting and Figure 2B shows the result in high transmission setting, assuming 85% coverage of the ACT (SP and artesunate). The dotted line shows the 50% resistance. Each curve represents the mean of ten simulations.

**Figure 3:**
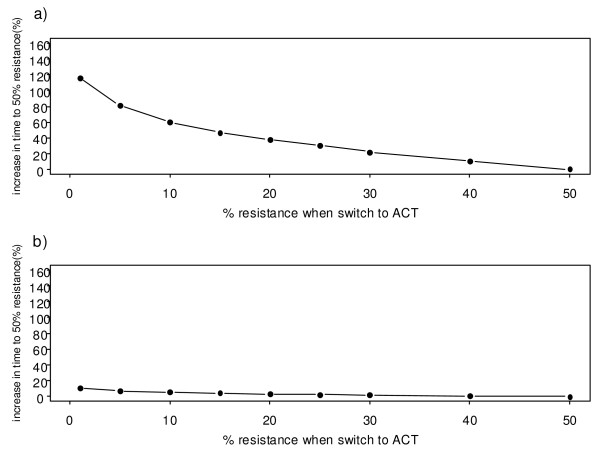
**Delay in the spread of resistance to SP**. The delay in the development of resistance is measured as the proportional increase in time to 50% resistance compared to the continued use of SP as a monotherapy.

In the first scenario, the model is used to predict the impact of varying the timing of switch to a high coverage (i.e. 85% of all symptomatic treated infections) by combining an artemisinin derivative with a failing partner drug (e.g. mefloquine), where the timing of the switch is governed by an observed prevalence of resistance to the partner drug in low and high transmission settings (Figure 2 and 3).

In the low transmission setting, treatment dependent parameter values are given in Table S8, Additional File 2. The force driving resistance comes from two sources; the first is from symptomatic malaria infections failing treatment and the second is exposure of infections to residual drug taken for presumptive or previous malaria treatment. More than 85% of all infections are symptomatic and thus treated. The rate of spread of resistance is faster relatively to the high transmission setting where treatment failure was identified to be the main force driving the spread of resistance. Deploying a high coverage of effective treatment, such as an ACT, when the level of resistance is still low delays the spread of drug resistance, a result consistent with previous results from simple epidemiological models [31].

In the high transmission settings, immunity prevents most acquired infections transmitting (and synergizes with anti-malarial drugs). Approximately 94% of all infections are asymptomatic and untreated. In the absence of anti-malarial treatment, the resistant infections have no survival advantage over the sensitive ones, and may have a fitness disadvantage. The main driving force for resistance is created from the selective filter provided by people carrying low residual concentrations of drugs, which protect against the establishment of new sensitive infections but not the resistant ones [32,33]. Without this residual drug effect, the rate of resistance would be much lower than shown in Figure 2B. Residual drug levels come mainly from previous 'presumptive' treatments (normally for other febrile illness), and are largely unrelated to the peaks of parasitaemia [9] and are, thereby, assumed to have little or no influence on the *de novo *resistance selection probability.

Combining an artemisinin with a drug to which resistance has arisen delays the spread of resistance in the low transmission setting (Figure 2A), but this delay decreases exponentially the later the switch is made to the ACT (Figure 3A). By contrast, in the high transmission setting, varying the time to switch to the ACT has only a small impact on delaying the spread of resistance (Figure 2B and 3B).

### Scenario B: Effects on artemisinin resistance of different levels of ACT coverage

**Figure 4:**
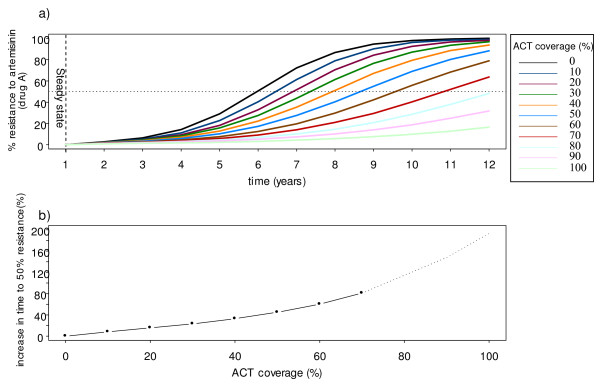
**The spread of artemisinin resistance at different levels of ACT coverage**. Figure 4A shows the spread of artemisinin resistance at varying levels of ACT coverage from 0% (i.e. use of artemisinin monotherapy) to 100% use of ACTs. Each line represents the mean of ten simulations. The dotted line shows the 50% resistance level. Figure 4B shows the delay in resistance measured as the increase in the time to 50% resistance (t_50_) compared with the t_50 _when using artemisinin monotherapy. The dotted line represents an extrapolation of the curve when the resistance does not reach 50% within the 12-year timeframe.

**Figure 5:**
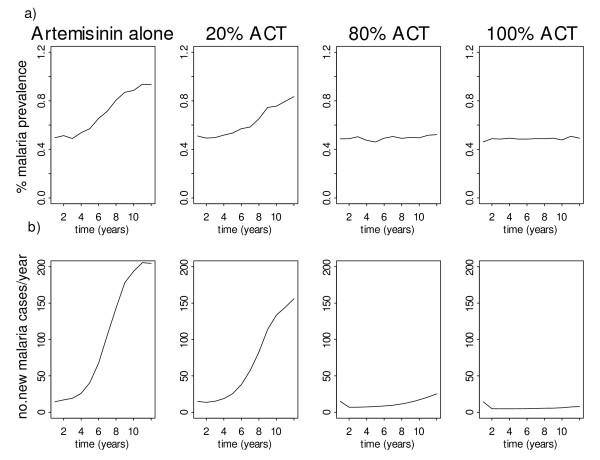
Change in malaria prevalence and incidence at different rates of coverage with ACT.

In this scenario, it is assumed that the monotherapy is the artemisinin and that drug resistance emerges to the artemisinin compound, rather than to the partner drug in an ACT (e.g. either piperaquine or lumefantrine), which is assumed to remain effective (see Table S8, Additional File 2). This simulates the current scenario in places such as Cambodia, where artesunate monotherapy use is widespread [34]. It is assumed that the switch to the combination therapy is made when the resistance to artemisinin is as low as 1% (Figure 4). If the switch is made very early, when there are still very few cases of drug resistance, then the higher the coverage with the ACT, the greater is the delay in the spread of resistance. At coverage rates of > 80%, the level of resistance to the artemisinin does not reach 50% within the time span of 10 years. In general, the impact of ACT deployment on malaria incidence and prevalence is as expected. By deploying ACTs at a high coverage, the prevalence of malaria can be kept at a very low level over time (0.5%) and incidence is less than 50 cases per year, indeed in the model eradication is only prevented by the influx of malaria in immigrants (Figure 5). Similar to the first scenario, the impact of ACT in the high transmission setting is much less.

The model shows that deploying ACT in a high transmission setting has a small impact on the spread of resistance and malaria prevalence. However, as one of the model outcomes, treatment failure in both low and high transmission settings can be sustained to a low level by deploying high ACT coverage (25% of recrudescence compared with 10% when deploying high ACT coverage at year 10). In order to make an impact on malaria transmission and resistance, vector control strategies need to be applied to reduce the VC. Reduction in the transmission intensity results in fewer infections, which, as host immunity declines, are more likely to be symptomatic and eventually this makes malaria control by drugs more effective.

## Discussion

**Figure 6:**
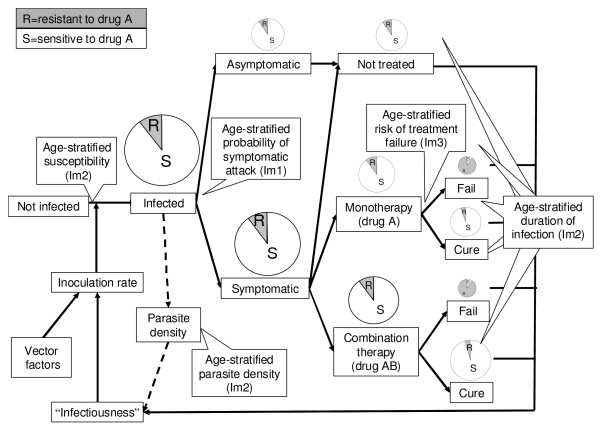
Schematic diagram of how immunity influences age-stratified likelihoods in the biological model.

A mathematical model always represents a simplified version of the true biological system. Complexity is traded against robustness. Malaria modeling has proven to be a useful tool for predicting the potential consequences of malaria control strategies [11,35]. In this paper, a model of suitable complexity to include all important features of malaria transmission and the spread of anti-malarial drug resistance in *P. falciparum *has been developed. The similarities between this and a prior simulation model by Cross are pronounced [36]. Both models looked far beyond the host population to the parasite and vector populations, however, this model has been modified to have the dynamic system for transmission and acquisition of host immunity (Figure 6).

The model predicts rapid spread of drug resistance in low transmission settings, and slower spread in high transmission settings. This is consistent with epidemiological observations. In low transmission settings a higher proportion of potentially transmissible infections are exposed to anti-malarial drugs and lower immunity increases the individual probability of treatment failure and transmission of resistant parasites. This prediction is also agreed by previous models [37-41]. The analysis in some of these models, however, pointed to the ecological explanation. For example, Koella and Antia (2003) described the maintaining low level of chloroqine resistance until the mid-70s could be due to the cost of resistance [41]. Similarly, in Klein *et al*. (2008), it was suggested that high level of clinical immunity create ecological refuge for drug-sensitive parasites. This implied that resistance is relatively easier to spread in a low transmission setting compared to a high transmission setting [40]. In low transmission settings, the spread of drug resistance can be slowed by combination treatments in which two or more effective drugs, which do not share resistance mechanisms, are combined. ACT is currently the combination of choice. This switch to combination treatment needs to be made early in the evolution of drug resistance with high rates of coverage (> 80%) if the full benefits in terms of delaying resistance are to be realized. This is the case whether the monotherapy to which resistance arises is an artemisinin derivative, or a non-artemisinin drug. The structure of this model allows many other policy relevant questions related to malaria control (vector controls, drug adherence and intermittent presumptive treatments, etc.) to be addressed. However, as a population based model, the ability to assess the effects of individual variation and the incorporation of important pharmacokinetic and pharmacodynamic variables is limited.

In low transmission settings, increasing ACT coverage is essential if the dramatic effects on malaria incidence observed recently in north-west Thailand, KwaZulu Natal and Zanzibar are to be extended to other areas [42,43]. *Plasmodium falciparum *malaria can be eliminated, although micro-heterogeneities in transmission intensity in remote areas will often ensure a protracted "end-game". The impact of ACT on drug resistance in high transmission intensity settings is limited because the majority of population is immune, many infections are asymptomatic, and therefore a smaller proportion of the infections are treated. An important weakness in the understanding of the epidemiology of malaria is the relative contribution of asymptomatic and symptomatic infections to transmission. The smaller the contribution of asymptomatic infections, the greater is the effect of ACT in slowing resistance spread. The main force driving the spread of drug resistance in these circumstances is the chemoprophylactic effect of presumptive therapy, which provides a selective filter for resistant parasites. The model also predicts that at low transmission intensities malaria transmission is readily eradicated without the continued influx of infected migrants. This has important implications for eliminating malaria [44,45].

The question of whether it is possible to reduce malaria transmission sufficiently to eliminate malaria eventually in high malaria transmission areas remains unresolved [8]. In high transmission settings, this model predicts that high ACT coverage alone cannot reduce malaria transmission unless it is used together with vector-control measures i.e. use of insecticides and deployment of insecticide-treated bed nets (ITN) and other materials to reduce the force of infection. To overcome the obstacles to high coverage of the un-affordability and un-availability of ACT, it has been argued persuasively that provision of global subsidies for co-formulated ACT must be provided at the top of the distribution chain. This would facilitate considerably the flow of drugs down to the end users through the existing public and private sector distribution pathways, with the ultimate objective of making effective anti-malarial treatments available and affordable even to the poorest people [46]. Stabilizing demand for ACT would also create incentives for ACT production, resulting in lower prices [7].

## Conclusion

A major obstacle to achieving the benefits of high coverage is the current cost of the drugs. The outputs provided by this model constitute a strong argument for a global subsidy to make ACT generally available and affordable in endemic areas. In addition, other interventions to reduce malaria transmission and to control usage of anti-malarial drugs may be required for slowing down the spread of drug resistance in high transmission intensity settings.

The spread of resistant parasites presents a very real public health risk and, in the absence of surveillance data, data-driven theoretical models provide the best information to assist policy making. This relatively complex model of malaria transmission and drug resistance provides a good framework for further development of the model to guide decision-making of other public health policies.

## Abbreviations

SP: sulphadoxine – pyrimethamine; ACT: artemisinin combination therapy; GSR: gametocyte switching rate, proportion of asexual parasites committing to sexual stage differentiation; EIR: entomological inoculation rate; VC: vectorial capacity; AIC: the Akaike information criterion; AUCgam: area under the curve of time versus blood gametocyte density; PRR: parasite reduction ratio, fractional reduction in parasitaemia per asexual cycle; LHS: Latin hypercube sampling; CV: coefficient of variation; PRCC: partial rank correlation coefficient; ITN: insecticide-treated bednet.

## Authors' contributions

WP developed and programmed the model and co-wrote the paper. SY developed the model, focusing on parameterization and co-wrote the paper. IH provided intellectual guidance on the mathematical modeling. AD provided suggestions on parasite dynamics and edited the paper. ND provided input on model design. NJW conceived of the study, provided overall intellectual guidance and edited the paper.

## Supplementary Material

Additional file 1 Model formalism. Click here for file

Additional file 2 Tables of parameters for sensitivity and scenario analyses. Click here for file

Additional file 3 Sensitivity analysis results. Click here for file
