# Supplementary material for: Spread of anti-malarial drug resistance: Mathematical model with implications for ACT drug policies
Source: Malar J. 2008 Nov 2;7:229. doi: 10.1186/1475-2875-7-229 (PMC2585590; doi:10.1186/1475-2875-7-229)
Supplement: Additional file 1 — Model formalism. [file 1475-2875-7-229-S1.doc]

**Supporting Information: Additional File 1 (Model formalism)**

**Spread of anti-malarial drug resistance: Mathematical model with implications for ACT drug policies**

Authors: Wirichada Pongtavornpinyo, Shunmay Yeung, Ian M Hastings, Arjen M Dondorp, Nicholas PJ Day, Nicholas J White

One iteration of the biological model is described as follow:

The model starts with the introduction of a specified number of new patent infections (“transmissible”) into an age-stratified population, which is assumed constant over time. The number of new infections at the beginning of an iteration is determined from the infectiousness of the population at the end of the previous iteration based on formula for the inoculation rate (*h*) given by Dietz [1]. The inoculation rate (*h*) is defined as the rate at which uninfected persons become infected and incorporates the rate of man-mosquito contact (VC), the proportion of humans that are potentially infectious and host’s susceptibility, which depends on the age-stratified level of immunity (Im2).

Dietz's formula is as follows:

(1)

where

*g* = the conditional probability that an infection results given at least one contact has occurred in a day or so called ‘host susceptibility’

VC = Vectorial Capacity

*Y_1_* = proportion of infected and infectious population. In this model, this is the product of the proportion of infected population and their mean infectiousness (*i*).

*n* = the duration of the extrinsic cycle of the parasites in the mosquito or the incubation period in mosquito

By assuming a Poisson distribution, the term gives the probability of having at least one contact per day. The inoculation rate is then simply the product of the host’s susceptibility (*g*) and this term.

In this model, the proportion of infections and infectiousness vary with resistance and treatment groups (i.e. among resistant/sensitive untreated, resistant/sensitive treated with monotherapy and resistant/sensitive treated with ACT groups). In order to track the rate of spread of resistance, the inoculation rate of resistant and sensitive infections is calculated separately. Thus, the proportion of infected and infectious sensitive infections called *Y_1s_* is the sum of all drug - sensitive infections treated with different treatments multiplied by their mean infectiousness (estimated from their average gametocyte density) over total number of infections i.e.

(2)

where

*T* = total number of treatment groups, *Y_s_* and *i* vary with treatment groups.

The proportion of infected and infectious drug - resistant infections called *Y_1r_* is the sum of all resistant infections treated with different treatments multiplied by their mean infectiousness over total number of infections i.e.

(3)

This yields two inoculation rates,

and (4)

The infectiousness of humans (*i*) is determined in part by the density of and duration for which mature gametocytes are present in circulation, and this can be expressed as the area under the gametocyte time curve. This, in turn, is dependent on the number of preceding asexual parasites, the Gametocyte Switching Rate (GSR), and the duration that mature gametocytes remain in circulation. The number of asexual parasites on any one day is dependent on the number of infected individuals, their age-stratified parasite density and the rate at which infections are cleared (the population recovery rate). In the model, gametocyte half-life was assumed to be 2.4 days (M6) [2]. For simplicity, the GSR was assumed to be uniform among asexual parasites and infections but to vary depending on the type of drug (S4) and between primary and recrudescent infections. As our understanding of the relationship between gametocyte dynamics, host immunity and infectiousness is still incomplete, we did not incorporate the direct effects of transmission blocking immunity in this model.

It is assumed that all humans are equally attractive to biting mosquitoes and mosquitoes are fully susceptible (V2) i.e. the infectiousness (*i*) to mosquitoes is assumed to be determined solely by the gametocyte density in humans. In order to calculate this relationship in this model, an exponential model was fitted to data from a study by Jeffery and Eyles, where mosquitoes were fed on blood from patients infected with different strains of *P.falciparum* [3]. This function is used at each iteration to calculate mosquito infectivity and is shown below.

*i* = (5)

The effect of residual drug from either presumptive treatment or previous malaria treatment is added on at this point in the model. The selective pressure on resistance is proportional to the fraction of the population harbouring residual drug. Although, the amount of residual drug and the degree of resistance determine selection (i.e. resistance spreads from hosts who have sufficient amount of residual drug to prevent establishment of the susceptible (or sensitive) form but unable to kill the tolerant (partial resistant) or fully resistant forms), we simply assume one level of residual drug which can prevent establishment of the new sensitive infections but not the new resistant infections. In future refinements to the model individual drug selection windows can be simulated at different transmission, treatment, pharmacokinetic and drug resistance characteristics and summed to provide population outcomes. Mathematically, the formula of the inoculation rate above (equation 4) is adjusted to:

(6)

(7)

where

*h_s_* is the rate at which the new drug-sensitive infection take place per day (i.e. inoculation rate of the drug-sensitive infections).

*h_r_* is the rate at which the new drug-resistant infection takes place per day (i.e. inoculation rate of the drug-resistant infections).

*RD* is the proportion of population with the residual drug, either from presumptive treatment or from previous malaria treatment, which can protect against the new drug-sensitive infections. Detailed calculations of the rate of spread of resistance from host populations with different amounts of residual drug and two levels of tolerance are described elsewhere [4,4].

All new patent infections are classified as either symptomatic or asymptomatic, and proportions depend on the age-stratified immunity (Im1). For patients who develop a symptomatic infection, the proportion receiving treatment is determined by the "treatment rate" and the proportion of those who receive a combination therapy (drug AB or BC) is determined by the "ACT coverage rate". Treated patients not receiving the combination therapy are assumed to receive the monotherapy (drug A). It is assumed that the likelihood, timing, and type of anti-malarial treatment received are independent of age (but age dependent differences determined by formulation availability, dose differences resulting from tablet size, cost, or behaviour could be introduced). As in other models, it is assumed that no treatment is given systematically to asymptomatic infections although the effects of residual drug levels on transmission are included as discussed above.

Treated infections may either be cured or the infection may recrudesce. By definition, recrudescence is more likely with “resistant” infections. The likelihood of recrudescence also depends on patient immunity, as well as the type of treatment and the level of patient adherence to therapy. Untreated infections resolve after a certain amount of time, depending on the setting for the maximum duration and the level of immunity. The model accommodates the possibility that a single infection can persist for many months untreated or partially treated [3,5,6].

At the end of each iteration, the infectiousness of the human population is re-estimated based on the number and distribution of gametocytes. The infectiousness and the input characteristics of the mosquito vector population are used to calculate the inoculation rate for the next iteration. The input vector characteristics can be varied to simulate different transmission intensities or vector control measures. This then closes the loop and begins the next iteration.

At the end of each daily iteration, the model outputs the total and age-group stratified number of patent infections, treated infections, cured and recrudescent infections, taking into account both new infections and pre-existing infections. Transmission intensity is also calculated in terms of annual Entomological Inoculation Rate (EIR) as a function of vectorial capacity (VC) which is given as input parameters in this model. EIR can be thought of as the human infecting rate. If *P* is the proportion of human population who are infected and their mean infectiousness to mosquitoes is *i*, the expected pool of infectiousness in the population is then equal to *Pi*. The EIR can then be formulated as:

EIR = *VCPi*365 (8)

where the factor of 365 converts the daily inoculation rate into an annual rate.

References

1. Dietz K, Molineaux L, Thomas A: **A malaria model tested in the African savannah.** *Bull World Health Organ* 1974, **50:**347-357.

2. Smalley ME, Sinden RE: ***Plasmodium falciparum* gametocytes: their longevity and infectivity.** *Parasitology* 1977, **74:**1-8.

3. Jeffery GM, Eyles DE: **Infectivity to mosquitoes of *Plasmodium falciparum* as related to gametocyte density and duration of infection.** *Am J Trop Med Hyg* 1955, **4:**781-789.

4. Hastings IM, Watkins WM: **Intensity of malaria transmission and the evolution of drug resistance.** *Acta Trop* 2005, **94:**218-229.

5. Collins WE, Jeffery GM: **A retrospective examination of the patterns of recrudescence in patients infected with *Plasmodium falciparum*.** *Am J Trop Med Hyg* 1999, **61:**44-48.

6. Eyles DE, Young MD: **The duration of untreated or inadequately treated *Plasmodium falciparum* infections in the human host.** *J Natl Malar Soc* 1951, **10:**327-336.
