# Supplementary material for: Spread of anti-malarial drug resistance: Mathematical model with implications for ACT drug policies
Source: Malar J. 2008 Nov 2;7:229. doi: 10.1186/1475-2875-7-229 (PMC2585590; doi:10.1186/1475-2875-7-229)
Supplement: Additional file 2 — Tables of parameters for sensitivity and scenario analyses. [file 1475-2875-7-229-S2.doc]

**Supporting Information: Additional File 2 (Parameters for sensitivity and scenario analyses)**

**Spread of anti-malarial drug resistance: Mathematical model with implications for ACT drug policies**

Authors: Wirichada Pongtavornpinyo, Shunmay Yeung, Ian M Hastings, Arjen M Dondorp, Nicholas PJ Day, Nicholas J White

**Tables of parameters for sensitivity and scenario analyses**

i) Order of presentation for parameter tables (Table S1 – S6)

The parameters are presented in the following order:

1. Malaria infection in the human host
2. Host immunity
3. Characteristics of asymptomatic infections
4. Characteristics of symptomatic infections
5. Characteristics of recrudescent infections
6. Vector dynamics

## ii) Rows and Columns

Each row in the tables represents a different parameter with information presented in 7 columns as shown below:

| 1. | 2. | 3. | 4. | 5. | 6. | 7. |
| --- | --- | --- | --- | --- | --- | --- |
| No. | Parameter | Dependent on | Influences | **Distribution + value in the model**  Reference values | Quality level (QL) | Assumptions and notes |
| M1 | Population age structure | Socio-economics  Migration | Distribution of infections  Levels of host immunity | **Fixed**  **African age structure**^1^ | A | Assume that the age structure is constant, unaffected by migration and fixed at the population size of 10,000. |

1. No. – Parameter number (to aid cross-referencing)
2. Parameter – Usually self-explanatory and if not then a brief definition is included
3. Dependent on – This indicates the factors that would affect this parameter.
4. Influences – This indicates the parameters “downstream” to this parameter.
5. **Distribution + value in the model** and Reference values – This is the distribution of parameter used in the sensitivity analysis e.g. uniform, normal or log-normal. For parameters for which there is little uncertainty a “fixed” input is used either as single value or as a vector e.g. age-stratified blood volume. The **bold** value shows the one used in the model while the other values are reference values obtained from the literature.
6. Quality level (QL) – This indicates the degree of confidence in the quality of the data and was defined as follows:

A Much data available, little uncertainty

B Some data available, some uncertainty

C Little or no data available, much uncertainty.

1. Assumptions and notes – Assumptions made for using the parameter in the model and useful information about the data.

Table S1: Malaria infection in the human host

| No. | Parameter | Dependent on | Influences | **Distribution + value in the model**  Reference values | QL | Assumptions and notes |
| --- | --- | --- | --- | --- | --- | --- |
| M1 | Population age structure | Socio-economics  Migration | Distribution of infections  Levels of host immunity | **Fixed**  **African age structure** [1] | A | Assumptions  The age structure is constant, unaffected by migration and fixed at the population size of 10,000.  There are three main age groups are considered i.e. 1 – 5, 6 – 12 and 12 – 60. Risk of malaria is uniformly distributed among the population.  Note  This can be adapted for atypical age structures e.g. in predominantly migrant populations. |
| M2 | Age-stratified blood volume per person | Age-weight structure of population | Calibration to obtain the actual number of parasites per infected person | **Fixed**  **0.8 – 5 litres**  Reference value  Blood volume is 75 ml/kg [2] | A | Assumptions  Average adult weight is 60kg for female and 65kg for male [3,4].  Blood volume of an adult aged 60 years old is 5 litres. |
| M3 | Time taken to acquire immunity after infection |  | Frequency that immunity profile of population is updated. | **Fixed**  **90 days** | C | Assumption  The time lag is the same for different facets of immunity.  Notes  Immunity time lag reflects loss and gain in immunity.  This parameter is not a biological parameter but a computational strategy to save computing time. |
| M4 | Pre-patent period or time taken from inoculation to detectable parasitaemia | Size of inoculation | Calculation of time lag between inoculations resulting in infectiousness | **Fixed**  **10 days**  Reference values  Pre-patent period = 11$\pm$ 0.16 days [5], 15 days [6], 11 (SD = 2.4) days [7] | A | Assumption  This period is not associated with host immunity. |
| M5 | Time from parasite patency to gametocyte patency (i.e. gametocyte maturation) |  | As for M4 | **Fixed**  **10 days** [8,9]  Reference values  Gametocyte circulation period of 6.4 days [10], gametocytogony’ (including a series of asexual cycles to sexual form) = 7 – 10 days [7] | A |  |
| M6 | Gametocyte half-life | Treatment [11] | Infectivity | **Fixed**  **2.4 days** [12]  Reference value  Mature gametocytes survive on average for 1.3 to 22.2 days (the geometric mean of 6.4 days) [10]. | A | Assumptions  Gametocytes once mature remain infective throughout their life.  Gametocytes clear at constant rate.  Notes  Average (geometric mean) circulation time after inoculation was 7.4 days - double that predicted by Smalley [10].  The effect of gametocytocidal drugs on gametocyte half-life is not considered. |
| M7 | Minimum parasite density detectable by microscopy | Microscopy techniques | Interpretation of prevalence data  Duration of infection  Minimum number of parasites when defining patent infection. | **Fixed**  **20/ul in thick film or 10^7^ parasites per adult** [13]  Reference value  Detectable parasite density = 5/ul [14] | A | Assumption  A white blood cell count of 8,000 per microliter of blood.  Note  Blood volume calibration is applied to the detectable limit. |
| M8 | Multiplication rate per 48 hours | Host immunity  Disease severity  Resistance | Duration and density of parasitaemia before it reaches a maximum | **Fixed**  **10**  Reference values  Multiplication rate of 8 with 90% prediction interval (5.5 – 12.3) [15], ranged between 6 – 10 [16], Ranged between 3 – 10 [17], ranged between 2.8 – 8.3 [18,19], 15 [20] | B | Assumption  Multiplication is unaffected by disease severity, resistance and host immunity. |
| M9 | Proportion of human population with inhibitory concentration of anti-malarials in their blood at any one time | Transmission intensity  Treatment seeking behaviour  Access to treatment | Likelihood that a sensitive infection will survive to become patent | **Uniform (0 – 40%)**  Reference values  18% of children with detectable SP while 5% of children with detectable CQN [21], 24.2% with two week recall of taking anti-malarial and positive test [22], 14% of pregnant women in 4 district of Kenya took SP when they have no malaria [23] | B |  |

Table S2: Host immunity

| No. | Parameter | Dependent on | Influences | **Immunity measure/Distribution and value in the model**  Reference values | QL | Assumptions and notes |
| --- | --- | --- | --- | --- | --- | --- |
| IM1 | Age-stratified probability of clinical symptoms in humans with a patent infection. | Transmission intensity | Used to construct Immunity Function 1 (Im1) | **Thailand (EIR ~ 1/year) range of 0.94 – 1.0** (Nosten F *et al.*, data file)**, Chonyi, Kenya (EIR ~ 50) range of 0 – 0.25** [24]**, Ngerenya, Kenya, (EIR ~ 20) range of 0 – 0.5** [24]**, Siaya, Kenya, (EIR ~ 270) range of 0.008 – 0.28** [25] | B | Note  Data from cross - sectional prevalence surveys. |
| IM2 | Age-stratified parasite density (parasites/uL) | Transmission intensity | Used to construct Immunity Function 2 (Im2) | **Ghana (EIR ~ 300) range of 117 – 1,922/uL** [26]**, Laos (EIR ~ 1) range of 5,024 – 97,591/uL** (Newton P *et al.*, data file)**, Kenya (EIR ~ 50) range of 40 – 439,179 /uL** [24]**, Kenya (EIR ~ 20) range of 40 – 694,629 /uL** [24]**, Thailand (EIR ~ 1) range of 40 – 1,528,753 /uL** (Nosten F *et al.*, data file)**, PNG (EIR ~ 40) range of 8,801– 55,228/uL** [27]**, Senegal (EIR ~ 200) range of 250 – 52,052 /uL** [28]**, Siaya, Kenya (EIR ~ 270) range of 500 – 4,500 /uL** [25] | A | Assumption  Detectable limit is 20/ul (=10^7^ parasites in adult).  Note  Data from cross - sectional prevalence surveys. |
| IM3 | Age-stratified risk of severe malaria | Transmission intensity | Used to construct Immunity Function 3 (Im3) | **Northern Kilifi, Kenya (EIR ~ 10), Siaya, Kenya (EIR ~ 100) and southern Kilifi (EIR ~ 120) range of 0 – 80 cases per 1,000 population, Sukuta, The Gambia (EIR ~ 2) range of 0 – 40 cases per 1,000 population, Bakau, The Gambia (EIR ~ 0.5) range of 0 – 10 cases/1,000 population (data from children aged below 9 years)** [29]**, Thailand (EIR ~ 1) range of 0 – 90 cases per 1,000 population (aged 0 to 10 years)** [30] | B | Note  Data from cohort studies and prevalence surveys. |
| IM4 | Maximum susceptibility i.e. maximum probability of developing a patent infection following inoculation |  | Used to calculate susceptibility to infection by age and transmission intensity | **Uniform (0.6 – 1)**  Reference value  0.78 non-immune adults became parasitaemic after first inoculation [31] | C | Note  After 1 inoculation, 71% of non-immune neurosyphilis patients became parasitaemic with fever and 7% parasitaemic without fever [31]. |
| IM5 | Maximum likelihood of treatment failure in sensitive infection treated with monotherapy | Response to treatment by individuals | Used to calculate the probability of treatment failure by age and transmission intensity | **Uniform (5 – 15%)**  Reference values  Maximum treatment failure in mefloquine- sensitive infections treated with mefloquine: 6.75% (see note), Indonesia 8.5% [32], Thailand 19% (by day 28) [33], Thailand 24% [34], Myanmar 1.7% for adults and 4.5% for children [35], Myanmar 7% [36,37], Thai-Cambodia border 27% [38].  Maximum treatment failure in SP – sensitive infections treated with SP: Mpumalanga; South Africa 6.4% (day 42) [39], Burkina Faso <1% [40],  Gambia 3.7% [41], Gambia 17.6% [42], East Sudan 0% [43] | A | Assumption  The maximum failure rate occurs in a non-immune person who has a resistant infection and is treated with monotherapy.  Notes  In the Thai study, artesunate was give at 10mg/kg for 1 – 2 days [44].  These data derive from clinical trials with > 28 days follow-up usually supported by genotyping.  Data from prospective studies supplemented by Delphi method (pooling of experts’ opinion). |
| IM6 | Maximum likelihood of treatment failure in resistant infection treated with monotherapy | Resistance mechanism as a result of mutation or amplification conferring reduced drug susceptibility |  | **Uniform (90 – 100%)**  Reference values  Maximum treatment failure in mefloquine-resistant infections treated with mefloquine: 60.8% by Delphi method, Thailand 49% [45].  Maximum treatment failure in SP-resistant infections treated with SP: Uganda 59.5% (by day 28) [46], Uganda 32% (by day 28) [47], Uganda 37% (by day 28) [48], Kenya 46% (by day 28) [49], Myanmar 35% [36], Myanmar 67% [37], Malawi 61 – 73% [50], Mozambique 21.4% (including RI, RII and RIII) [51] | B |  |
| IM7 | Relative likelihood of treatment failure in sensitive infection treated with ACT | Maximum likelihood of treatment failure in sensitive infections treated with monotherapy (IM5) |  | **Uniform (0.1 – 0.5), *giving the actual treatment failure of sensitive infections treated with ACT = 0.5 – 7.5%***  Reference values  Maximum treatment failure in mefloquine-sensitive infections treated with mefloquine + artesunate: Laos 0% (by day 42) [52], Thailand 0% (by day 28) [33], Thailand 2% [34], Cambodia 5% (Yeung S, personal communication).  Maximum treatment failure in SP-sensitive infections treated with SP + artesunate: Gambia 0% [53], Gambia 5% [42] | B |  |
| IM8 | Relative likelihood of treatment failure in resistant infection treated with ACT | Maximum likelihood of treatment failure in resistant infection treated with monotherapy (IM6) |  | **Uniform (0.1 – 0.5), *giving the treatment failure of resistant infection treated with ACT = 9 – 50%***  Reference values  Maximum treatment failure in SP-resistant infections treated with SP + artesunate (3 days): Uganda 26% (by day 28) [46], Uganda 17% (by day 8) [47], Kenya 26% (by day 28) [49] | B |  |
| IM9 | Maximum probability of symptoms in a patent infection |  |  | **Uniform (0.6 – 1)**  Reference values  Maximum probability of being symptomatic = Uganda 0.89 (in < 5 years old) [54], 0.95 [31], Thailand 1 (Nosten F *et al.*, data file), Thailand 0.84 – 0.93 [55], Kenya 0.28 [56], Kenya 0.25 **–** 0.50 [24], Sudan 0.5 [57] | B | Note  This parameter was used to start the iterative model when the immunity profile was initially unknown. |
| IM10 | Maximum duration of untreated infection |  | Influences population rate of loss of infections  Influences time before individual re-enters susceptible pool or starts recrudescent infection | **Lognormal (mean = 80, SD = 1.2), *giving the actual duration between 40 – 150 days***  Reference values  Duration of untreated infection = 121 $\pm$ 9 days [9],  147 days [58]**,** 75% of all infections lasted up to 2 months and none by 3 months [5], 9.5 months [59,60], 52 – 588 days (derived value) [59], 40 weeks for single genotype and less than 4 weeks in first two years of life [61], greater than 48 days for less than 4 years of age, 9 days for age 5 – 9 years, 15 days for age 10 – 14 years, 12 days for adults [62], more than 18 months [63]**,** 200 – 300 days [64], 152 days (model estimate) [65] | B |  |
| IM11 | Parasite reduction ratios (PRR) of sensitive infection treated with monotherapy | Drug  Resistance | Used to derive duration of infections and rate at which parasites are cleared from the population | **Uniform (500-1500) fold reduction per life cycle**  Reference value  PRR of mefloquine or SP ranged from 10 – 1,000 [66] | A | Assumptions  Reduction in parasite population is log-linear [67].  Treatment is given at maximum parasitaemia. |
| IM12 | PRR of resistant infection treated with monotherapy |  |  | **Uniform** (**50 – 150) fold reduction per life cycle**  Reference values  PRR of quinine, mefloquine or SP in Thai-Cambodian border is less than 100 in sensitive infections and 25 in resistant infections [66,68], PRR of mefloquine in Thai-Cambodian border is more than 1000 for sensitive infection and less than 1000 for resistant infections [69], PRR for mefloquine in Thailand is less than 50 in resistant infections [45,70] | A | Same as IM11 |
| IM13 | Relative PRR of infection treated with ACT | PRR of infection treated with monotherapy (IM11 and IM12) |  | **Uniform (10 – 90), *giving the estimate PRR of ACT = 5,000 – 135,000***  Reference values  PRR of artesunate is ranged from 10^3^ to 10^5^ [66], 10^8^ [34] |  | Assumption  There is no resistance to ACT. |

Table S3: Characteristics of asymptomatic infections

| No. | Parameter | Dependent on | Influences | **Distribution + value in the model**  Reference values | QL | Assumptions and notes |
| --- | --- | --- | --- | --- | --- | --- |
| A1 | Relative parasite density of asymptomatic infection | Parasite density in symptomatic infections (IM2) | Infectiousness of untreated “immune” patients | **Uniform (0.1 – 0.9) *i.e. 10 – 90% of parasite density in symptomatic infections***  Reference values  Vauatu (EIR ~ 7) range of 0.01 – 0.03 [71], PNG 0.744 [72], Mali 0.55 [73], Ghana 0.7 [74] | A | Assumption  This ratio is fixed across age groups. |
| A2 | Relative gametocyte switching rate (GSR) (probability of an asexual parasite switching to a gametocyte) | GSR of an infection treated with monotherapy (S4) | Infectiousness of untreated “immune” patients | **Lognormal (mean = 1, SD = 1.7), *giving the actual range of 0.1 – 8* *i.e. switch rate is 10%of those treated to 8 times higher than those treated***  Reference values  GSR of 0.0019 in “acute” cases, 0.019 in “chronic” cases [8], 0.64 (range of 0.00027 – 0.135) [10] | B | Note  The calculation of the GSR is based on the ratio of the peak gametocyte to the peak asexual parasite. |

Table S4: Characteristics of symptomatic infections

| No. | Parameter | Dependent on | Influences | **Distribution + value in the model**  Reference values | QL | Assumptions and notes |
| --- | --- | --- | --- | --- | --- | --- |
| S1 | Treatment rate  (Probability that a symptomatic infection will be treated with an anti-malarial) | Access to treatment  Treatment seeking behaviour | Likelihood of cure, duration of infection and GSR | **Uniform (90 – 100%)**  Reference values  Treatment rate review ranged from 40 – 93% [75], Kenya 96% [76], Uganda 93% [77], Ethiopia 80% [78], South Africa 92 – 95% (Barns K, personal communication), Tanzania 84% [21], Philippines 80% [79] | A | Note  Patients receive either monotherapy (drug A) or combination therapy (drug AB or BC). |
| S2 | Coverage rate with ACT  (Likelihood that a treated patient receives ACT) | Access to treatment  Treatment seeking behaviour | Likelihood of cure, duration of infection and GSR | **Fixed (but varied between 0 – 1 in scenario analysis)**  Reference values  Cambodia with ACT coverage of 0.08 – 0.9 [80], South Africa 0.97 – 0.99 (Barns K, personal communication) | A | Note  Patients not covered with ACT receive monotherapy. |
| S3 | Duration of peak parasitaemia | Access to treatment  Treatment seeking behaviour | Duration of parasitaemia and therefore infectiousness | **Lognormal (mean = 1.2, SD = 1.2), *giving the actual range of 0.5 – 2.5 days***  Reference values  Delay in treatment in Ethiopia 1 – 2 days (43%), 3 – 4 days (31%) [78], West Kenya 0.6 $\pm$ 0.8 days [81], Thailand 2 days (s.d.0.9) [82], South Africa 4 days (Barns K, personal communication) | A |  |
| S4 | Gametocyte switching rate (GSR) of an infection treated with monotherapy | Drug (see detail in Table S8) | Infectiousness of treated patients | **Uniform (0.001 – 0.005)**  Reference values  Chloroquine GSR ~ 0.001 [11,83,84], 0.0002 [85], chloroquine-sensitive GSR ~ 0.00004, chloroquine-resistant GSR ~ 0.00054 [85]  SP GSR ~ 0.002 [85] | B | Assumption  GSR is independent of immunity.  Note  GSR was calculated by dividing the peak gametocyte density on day 7 by the trophozoite density on admission [85]. |
| S5 | Relative GSR of an infection treated with ACT | GSR of an infection treated with monotherapy (S4) |  | Uniform (0.1 – 0.5), *giving the estimate GSR for ACT is thus 0.0001 – 0.0025*  Reference values  Gametocyte carriage rate in the mefloquine group was much greater compared with the mefloquine + artesunate group i.e. the relative risk (RR) = 8 [86], 3-day artesunate reduced gametocyte on day 7 [87], chloroquine + artesunate gave 6 to 15 folds lower in oocyst numbers in membrane-fed mosquitoes when compared with chloroquine alone [83] | B |  |

Table S5: Characteristics of recrudescent infections

| No. | Parameter | Dependent on | Influences  Role in model | **Distribution + value in the model**  Reference values | QL | Assumptions and notes |
| --- | --- | --- | --- | --- | --- | --- |
| R1 | Relative infectiousness of recrudescent infections compared to primary infections | Parasitaemia in primary infection (IM2) | Used to check GSR in recrudescent infection | **Lognormal** **(mean = 4)**  Reference values  Duration of gametocytaemia was 4 times longer in recrudescence compared with primary infection [86], AUCgam in chloroquine-resistant infections was 14 times greater compared to chloroquine sensitive infections [11] | C | Note  The infectiousness of recrudescence is influenced by its GSR, duration of recrudescent infection and parasite density. |
| R2 | Relative mean parasitaemia in recrudescent infection | Parasitaemia in primary infection (IM2) | Infectivity of resistant infections | **Uniform (0.1 – 0.9) , *giving the mean parasitaemia in recrudescence of 10 – 90% of those in primary infections***  Reference values  For chloroquine recrudescent infection ~ 0.1 [11], 0.71 (range of 0.002 – 3.96) (Barns K, personal communication), Sri Lanka chloroquine ~ 0.28, primaquine ~ 0.22 [84], 4 – 7times less in non-immune non-treated [58] | B |  |
| R3 | Relative gametocyte switching rate in recrudescent infection | GSR of a treated infection (S4, S5) | Infectivity of resistant infections | **Lognormal (mean = 20, SD = 1.2), *giving the actual range between 10 and 40 times higher in GSR in recrudescent infections***  Reference values  For chloroquine ~ 52.6 (i.e. GSR of 0.051 in recrudescence versus GSR of 0.00097 in primary infection) [11] | C |  |
| R4 | Time interval between disappearance of parasites in the initial infection and reappearance of parasites in recrudescent infections | Immunity  Degree of resistance  Half-life of anti-malarial drug | Infectivity of resistant infections  Duration of recrudescent infection | Normal (mean = 14, SD = 2) for SP, *giving the actual range of 5 – 21 days*  Reference values  Mean time to recrudescence for SP ~ 28 days (White N, personal communication), Time to recrudescence ~ 24 – 33 days depending on drug half life [88] | B | Note  Estimated from the time between treatment of initial infection and peak parasitaemia in recrudescent infection i.e. time to recrudescence. |
| R5 | Number of recrudescence | Treatment received | Infectivity of resistant infections | **Fixed**  **3 peaks**  Reference values  In non-immune neurosyphilis patients, 1 – 5 peaks after which they are” barely detectable” [89] | B | Assumption  The number of recrudescence is independent of immunity. |

Table S6: Vector dynamics

| No. | Parameter | Dependent on | Influences | **Distribution + value in the model**  Reference values | QL | Assumptions and notes |
| --- | --- | --- | --- | --- | --- | --- |
| V1 | Vectorial capacity (VC) | Human biting rate  Anopheles survival  Vector control | Inoculation rate | **Uniform (low = 0.1, high = 15)**  Reference values  Thailand 0.48 – 1.28 dry season [90], Tanzania with *An. Arabiensis* 0.34 – 1.42 [91], Laos with *An. Dirus* 0.009 – 0.43 [92], India with *An Dirus* 0 – 0.82 [93], Nigeria with *An. Gambiae* 15 [94], VC reviewed from 159 sites in 15 countries [95] | C | Note  VC is particularly sensitive to duration of Anopheles survival. |
| V2 | Likelihood that a mosquito will be infected after biting a human carrying gametocytes | Gametocyte density in humans  Packed cell volume | Use to check the mosquito infectivity calculated by the model | **Fixed**  **0.15-1**  Reference values  For gametocyte density < 10/ul, the probability of infecting mosquito = 0.21, for gametocyte density >1000/ul = 1 [96], Tanzania by membrane fed, mean probability = 0.25 and maximum = 0.77 [97], Cameroon by direct fed, mean probability = 0.19 [98], PNG 0.15$\pm$ 0.029 [99], The Gambia 0.58 [100], Tanzania 0.42 [100] | A | Assumption  This is not affected by host immunity [96].  Note  In the model, this parameter is calculated by (equation 5) within model iterations. |
| V3 | Duration of sexual stage in mosquito | Vector type | Time lag of between human infections | **Fixed**  **12 days**  Reference values  11 days in summer and 28 days in winter [6] | A | Assumption  In the model, this duration is fixed and independent of seasonality. |

Table S7: **Facets of immunity and data used in the model**

| **Facet of immunity** | **Explanation** | **Age and EIR stratified data used in immunity function** | **Immunity function (Im)** | **Parameter for “zeroing” immunity function** |
| --- | --- | --- | --- | --- |
| Reduction in symptomatic disease | Reduction in the likelihood that a patent infection will be symptomatic (“antitoxic immunity”) | Likelihood of symptoms during infection | Im1 | Direct function i.e. required no parameter for zeroing |
| Reduction in “Susceptibility” | Reduction of the probability that an inoculation becomes a detectable and therefore potentially transmissible infection (pre-erythrocytic or liver stage and blood stage* immunity) | Mean parasite density | Im2 | Maximum susceptibility of a non-immune host |
| Reduction in maximum parasite density | Reduction in the probability that viable merozoites released from the liver will multiply during the blood stage of infection to reach high densities (blood stage immunity*) | Mean parasite density | Im2 | Maximum parasite density in non-immune host |
| Reduction in the duration of infection | Increased rate of clearance of parasites (blood stage immunity*) | Mean parasite density | Im2 | Maximum durations of different types of infection in non-immune host |
| Increase in self-cure and cure rate | Increased clearance of parasites and therefore likelihood of self or drug induced cure (blood stage immunity*) | Risk of severe malaria | Im3 | Maximum failure rate in different types of infection in non-immune host |
| Reduction in severe malaria or death | Reduction in likelihood that a symptomatic infection will become severe (blood stage immunity*) | Not included in the model but incorporated into cost and effectiveness analysis | | |
| Reduction in transmissibility of infection | Reduction in the viability and transmissibility of formed gametocytes (in addition to increased clearance of parasites) (transmission blocking immunity) | Not included directly in the model | | |

* Blood stage immunity: the dynamics of the blood stage immunity contribution to these effects differs for each facet.

**Immunity functions:**

$P$(being symptomatic) = $0.13\exp^{-0.5AGE}+0.92\exp^{-0.12EIR}+0.15\exp^{-0.002AGE\times EIR}$ (Im1)

log_10_(parasite) = $9.8\exp^{-0.001AGE+0.000003EIR-0.000002AGE\times EIR}+0.97\exp^{0.011AGE+0.003EIR-0.0045AGE\times EIR}$ (Im2)

$P$(severe malaria) = $1.28\exp^{-0.59AGE-0.003EIR}+0.63\exp^{-0.04AGE-0.11EIR}$ (Im3)

**Table S8: Table of parameters for scenario analysis**

**The parameter values describe the characteristics of monotherapy and artemisinin based combination therapy (ACT) used in the model**

| **Parameter** | **Values used in Scenario A** | | **Values used in Scenario B** | |
| --- | --- | --- | --- | --- |
|  | **SP alone** | **Artemisinin and SP** | **Artemisinin alone** | **Artemisinin +Lumefantrine or Piparaquine** |
| PRR* of drug-sensitive infections | 1,000  Uniform (500 – 1,500) | 50,000  Uniform (5,000 – 135,000) | 50,000 | 50,000 |
| PRR of drug-resistant infections | 100  Uniform (50 – 150) | 5,000  Uniform (500 – 13,500) | 1,000 | 1,000 |
| Failure rate in drug-sensitive infections | 10%  Uniform (5 – 15) | 3%  Uniform (1 – 7.5) | 10 | 10 |
| Failure rate in drug-resistant infections | 95%  Uniform (90 – 100) | 28.5%  Uniform (9 – 50) | 95% | 20% |
| GSR** | 0.003  Uniform (0.001 – 0.005) | 0.0009  Uniform (0.0001 – 0.0025) | 0.0009 | 0.0009 |
| Time interval between disappearance of parasites in the initial infection and reappearance of parasites in recrudescent infections (days) | Normal (mean = 14, SD = 2) | Normal (mean = 14, SD = 2) | 10 | 19 |
| Proportion of human population with significant residual drug levels | 10%  (SP) | 10%  (SP) | 0%  (artemisinin) | 0%  (artemisinin) |

*PRR = Parasite reduction ratio

**GSR = Gametocyte switching rate

References

1. United Nations. **African's population structure.** 2000. [http://esa.un.org/unpp]

2. Diem K, Lentner C: *Scientific Tables.* Basle: 1970.

3. The National Center for Health Statistics. **Stature for age and weight for age percentiles.** The National Center for Chronic Disease Prevention and Health Promotion . 21-11-2000.

4. WHO. **Standard Growth Chart for the world's population.** World Health Organisation . 9-9-2003. [http://www.who.int]

5. Kitchen SF: **Falciparum Malaria.** In *Malariology*. Edited by Edited by Boyd MF. Philadelphia: Saunders; 1949:995-1016.

6. Dietz K, Molineaux L, Thomas A: **A malaria model tested in the African savannah.** *Bull World Health Organ* 1974, **50:**347-357.

7. White NJ: **Malaria.** In *Manson's Tropical Diseases*. 21 edition. Edited by Edited by Cook GC, Zumla A. London: Saunders; 2003:1205-1295.

8. Thomson D.: **A research into the production, life and death of crescents in malignant tertian malaria, in treated and untreated cases by and enumerative method.** *Annals of Tropical Medcine and Parasitology* 1911,57-85.

9. Eyles DE, Young MD: **The duration of untreated or inadequately treated *Plasmodium falciparum* infections in the human host.** *J Natl Malar Soc* 1951, **10:**327-336.

10. Eichner M, Diebner HH, Molineaux L, Collins WE, Jeffery GM, Dietz K: **Genesis, sequestration and survival of *Plasmodium falciparum* gametocytes: parameter estimates from fitting a model to malariatherapy data.** *Trans R Soc Trop Med Hyg* 2001, **95:**497-501.

11. Sowunmi A, Fateye BA: ***Plasmodium falciparum* gametocytaemia in Nigerian children: before, during and after treatment with antimalarial drugs.** *Trop Med Int Health* 2003, **8:**783-792.

12. Smalley ME, Sinden RE: ***Plasmodium falciparum* gametocytes: their longevity and infectivity.** *Parasitology* 1977, **74:**1-8.

13. Bruce-Chwatt LJ: **History of malaria from prehistory to eradication.** In *Malaria: Principles and Practice of Malariology*. Edited by Edited by Werndorfer WH, McGregor I. Edinburgh: Churchill Livingstone; 1988:1-59.

14. Petersen E, Hogh B, Marbiah NT, David K, Hanson AP: **Development of immunity against *Plasmodium falciparum* malaria: clinical and parasitologic immunity cannot be separated.** *J Infect Dis* 1991, **164:**949-953.

15. Simpson JA, Aarons L, Collins WE, Jeffery GM, White NJ: **Population dynamics of untreated *Plasmodium falciparum* malaria within the adult human host during the expansion phase of the infection.** *Parasitology* 2002, **124:**247-263.

16. White NJ, Krishna S: **Treatment of malaria: some considerations and limitations of the current methods of assessment.** *Trans R Soc Trop Med Hyg* 1989, **83:**767-777.

17. White NJ, Chapman D, Watt G: **The effects of multiplication and synchronicity on the vascular distribution of parasites in falciparum malaria.** *Trans R Soc Trop Med Hyg* 1992, **86:**590-597.

18. Chotivanich K, Udomsangpetch R, Simpson JA, Newton P, Pukrittayakamee S, Looareesuwan S, White NJ: **Parasite multiplication potential and the severity of Falciparum malaria.** *J Infect Dis* 2000, **181:**1206-1209.

19. Gravenor MB, McLean AR, Kwiatkowski D: **The regulation of malaria parasitaemia: parameter estimates for a population model.** *Parasitology* 1995, **110 ( Pt 2):**115-122.

20. Fairley NH: **Sidelights on malaria in man obtained by sub-Inoculation experiments.** *Trans R Soc Trop Med Hyg* 1947, **40:**621-676.

21. Eriksen J, Nsimba SE, Minzi OM, Sanga AJ, Petzold M, Gustafsson LL, Warsame MY, Tomson G: **Adoption of the new antimalarial drug policy in Tanzania--a cross-sectional study in the community.** *Trop Med Int Health* 2005, **10:**1038-1046.

22. Talisuna AO, Langi P, Bakyaita N, Egwang T, Mutabingwa TK, Watkins W, Van Marck E, D'Alessandro U: **Intensity of malaria transmission, antimalarial-drug use and resistance in Uganda: what is the relationship between these three factors?** *Trans R Soc Trop Med Hyg* 2002, **96:**310-317.

23. Guyatt HL, Noor AM, Ochola SA, Snow RW: **Use of intermittent presumptive treatment and insecticide treated bed nets by pregnant women in four Kenyan districts.** *Trop Med Int Health* 2004, **9:**255-261.

24. Mwangi TW, Ross A, Snow RW, Marsh K: **Case Definitions of Clinical Malaria under Different Transmission Conditions in Kilifi District, Kenya.** *J Infect Dis* 2005, **191:**1932-1939.

25. Bloland PB, Boriga DA, Ruebush TK, McCormick JB, Roberts JM, Oloo AJ, Hawley W, Lal A, Nahlen B, Campbell CC: **Longitudinal cohort study of the epidemiology of malaria infections in an area of intense malaria transmission II. Descriptive epidemiology of malaria infection and disease among children.** *Am J Trop Med Hyg* 1999, **60:**641-648.

26. Owusu-Agyei S, Smith T, Beck HP, Amenga-Etego L, Felger I: **Molecular epidemiology of *Plasmodium falciparum* infections among asymptomatic inhabitants of a holoendemic malarious area in northern Ghana.** *Trop Med Int Health* 2002, **7:**421-428.

27. Cox MJ, Kum DE, Tavul L, Narara A, Raiko A, Baisor M, Alpers MP, Medley GF, Day KP: **Dynamics of malaria parasitaemia associated with febrile illness in children from a rural area of Madang, Papua New Guinea.** *Trans R Soc Trop Med Hyg* 1994, **88:**191-197.

28. Trape JF, Rogier C, Konate L, Diagne N, Bouganali H, Canque B, Legros F, Badji A, Ndiaye G, Ndiaye P: **The Dielmo project: a longitudinal study of natural malaria infection and the mechanisms of protective immunity in a community living in a holoendemic area of Senegal.** *Am J Trop Med Hyg* 1994, **51:**123-137.

29. Snow RW, Omumbo JA, Lowe B, Molyneux CS, Obiero JO, Palmer A, Weber MW, Pinder M, Nahlen B, Obonyo C, Newbold, C, Gupta, S, Marsh, K: **Relation between severe malaria morbidity in children and level of *Plasmodium falciparum* transmission in Africa.** *Lancet* 1997, **349:**1650-1654.

30. Luxemburger C, Ricci F, Nosten F, Raimond D, Bathet S, White NJ: **The epidemiology of severe malaria in an area of low transmission in Thailand.** *Trans R Soc Trop Med Hyg* 1997, **91:**256-262.

31. Ciuca M, BalliF L, Chelarescu-Vieru M: **Immunity in malaria.** *Trans R Soc Trop Med Hyg* 1934, **27:**619-622.

32. Sutanto I, Supriyanto S, Ruckert P, Purnomo, Maguire JD, Bangs MJ: **Comparative efficacy of chloroquine and sulfadoxine-pyrimethamine for uncomplicated *Plasmodium falciparum* malaria and impact on gametocyte carriage rates in the East Nusatenggara province of Indonesia.** *Am J Trop Med Hyg* 2004, **70:**467-473.

33. Looareesuwan S, Viravan C, Vanijanonta S, Wilairatana P, Suntharasamai P, Charoenlarp P, Arnold K, Kyle D, Canfield C, Webster K: **Randomised trial of artesunate and mefloquine alone and in sequence for acute uncomplicated falciparum malaria.** *Lancet* 1992, **339:**821-824.

34. Simpson JA, Price R, ter Kuile F, Teja-Isavatharm P, Nosten F, Chongsuphajaisiddhi T, Looareesuwan S, Aarons L, White NJ: **Population pharmacokinetics of mefloquine in patients with acute falciparum malaria.** *Clin Pharmacol Ther* 1999, **66:**472-484.

35. Tin F, Hlaing N, Lasserre R: **Single-dose treatment of falciparum malaria with mefloquine: field studies with different doses in semi-immune adults and children in Burma.** *Bull World Health Organ* 1982, **60:**913-917.

36. Ejov MN, Tun T, Aung S, Sein K: **Response of falciparum malaria to different antimalarials in Myanmar.** *Bull World Health Organ* 1999, **77:**244-249.

37. Smithuis FM, Monti F, Grundl M, Oo AZ, Kyaw TT, Phe O, White NJ: ***Plasmodium falciparum*: sensitivity *In vivo* to chloroquine, pyrimethamine/sulfadoxine and mefloquine in western Myanmar.** *Trans R Soc Trop Med Hyg* 1997, **91:**468-472.

38. Smithuis FM, van Woensel JB, Nordlander E, Vantha WS, ter Kuile FO: **Comparison of two mefloquine regimens for treatment of *Plasmodium falciparum* malaria on the northeastern Thai-Cambodian border.** *Antimicrob Agents Chemother* 1993, **37:**1977-1981.

39. Mabuza A, Govere J, Durrheim D, Mngomezulu N, Bredenkamp B, Barnes K, Sharp B: **Therapeutic efficacy of sulfadoxine-pyrimethamine in uncomplicated *Plasmodium falciparum* malaria 3 years after introduction in Mpumalanga.** *S Afr Med J* 2001, **91:**975-978.

40. Tinto H, Zoungrana EB, Coulibaly SO, Ouedraogo JB, Traore M, Guiguemde TR, Van Marck E, D'Alessandro U: **Chloroquine and sulphadoxine-pyrimethamine efficacy for uncomplicated malaria treatment and haematological recovery in children in Bobo-Dioulasso, Burkina Faso during a 3-year period 1998-2000.** *Trop Med Int Health* 2002, **7:**925-930.

41. von Seidlein L, Bojang K, Jones P, Jaffar S, Pinder M, Obaro S, Doherty T, Haywood M, Snounou G, Gemperli B, Gathmann I, Royce C, McAdam K, Greenwood B: **A randomized controlled trial of artemether/benflumetol, a new antimalarial and pyrimethamine/sulfadoxine in the treatment of uncomplicated falciparum malaria in African children.** *Am J Trop Med Hyg* 1998, **58:**638-644.

42. Doherty JF, Sadiq AD, Bayo L, Alloueche A, Olliaro P, Milligan P, von Seidlein L, Pinder M: **A randomized safety and tolerability trial of artesunate plus sulfadoxine--pyrimethamine versus sulfadoxine-pyrimethamine alone for the treatment of uncomplicated malaria in Gambian children.** *Trans R Soc Trop Med Hyg* 1999, **93:**543-546.

43. Adam I, Osman ME, Elghzali G, Ahmed GI, Gustafssons LL, Elbashir MI: **Efficacies of chloroquine, sulfadoxine-pyrimethamine and quinine in the treatment of uncomplicated, *Plasmodium falciparum* malaria in eastern Sudan.** *Ann Trop Med Parasitol* 2004, **98:**661-666.

44. Bunnag D, Viravan C, Looareesuwan S, Karbwang J, Harinasuta T: **Clinical trial of artesunate and artemether on multidrug resistant falciparum malaria in Thailand. A preliminary report.** *Southeast Asian J Trop Med Public Health* 1991, **22:**380-385.

45. ter Kuile FO, Luxemburger C, Nosten F, Thwai KL, Chongsuphajaisiddhi T, White NJ: **Predictors of mefloquine treatment failure: a prospective study of 1590 patients with uncomplicated falciparum malaria.** *Trans R Soc Trop Med Hyg* 1995, **89:**660-664.

46. Priotto G, Kabakyenga J, Pinoges L, Ruiz A, Eriksson T, Coussement F, Ngambe T, Taylor WR, Perea W, Guthmann JP, Olliaro P, Legros D: **Artesunate and sulfadoxine-pyrimethamine combinations for the treatment of uncomplicated *Plasmodium falciparum* malaria in Uganda: a randomized, double-blind, placebo-controlled trial.** *Trans R Soc Trop Med Hyg* 2003, **97:**325-330.

47. Dorsey G, Gasasira AF, Machekano R, Kamya MR, Staedke SG, Hubbard A: **The impact of age, temperature, and parasite density on treatment outcomes from antimalarial clinical trials in Kampala, Uganda.** *Am J Trop Med Hyg* 2004, **71:**531-536.

48. Checchi F, Piola P, Kosack C, Ardizzoni E, Klarkowski D, Kwezi E, Priotto G, Balkan S, Bakyaita N, Brockman A, Guthmann JP: **Antimalarial efficacy of sulfadoxine-pyrimethamine, amodiaquine and a combination of chloroquine plus sulfadoxine-pyrimethamine in Bundi Bugyo, western Uganda.** *Trop Med Int Health* 2004, **9:**445-450.

49. Obonyo CO, Ochieng F, Taylor WR, Ochola SA, Mugitu K, Olliaro P, ter Kuile F, Oloo AJ: **Artesunate plus sulfadoxine-pyrimethamine for uncomplicated malaria in Kenyan children: a randomized, double-blind, placebo-controlled trial.** *Trans R Soc Trop Med Hyg* 2003, **97:**585-591.

50. Plowe CV, Kublin JG, Dzinjalamala FK, Kamwendo DS, Mukadam RA, Chimpeni P, Molyneux ME, Taylor TE: **Sustained clinical efficacy of sulfadoxine-pyrimethamine for uncomplicated falciparum malaria in Malawi after 10 years as first line treatment: five year prospective study.** *BMJ* 2004, **328:**545.

51. Abacassamo F, Enosse S, Aponte JJ, Gomez-Olive FX, Quinto L, Mabunda S, Barreto A, Magnussen P, Ronn AM, Thompson R, Alonso PL: **Efficacy of chloroquine, amodiaquine, sulphadoxine-pyrimethamine and combination therapy with artesunate in Mozambican children with non-complicated malaria.** *Trop Med Int Health* 2004, **9:**200-208.

52. Mayxay M, Khanthavong M, Lindegardh N, Keola S, Barends M, Pongvongsa T, Yapom R, Annerberg A, Phompida S, Phetsouvanh R, White NJ, Newton PN: **Randomized comparison of chloroquine plus sulfadoxine-pyrimethamine versus artesunate plus mefloquine versus artemether-lumefantrine in the treatment of uncomplicated falciparum malaria in the Lao People's Democratic Republic.** *Clin Infect Dis* 2004, **39:**1139-1147.

53. von Seidlein L, Jawara M, Coleman R, Doherty T, Walraven G, Targett G: **Parasitaemia and gametocytaemia after treatment with chloroquine, pyrimethamine/sulfadoxine, and pyrimethamine/sulfadoxine combined with artesunate in young Gambians with uncomplicated malaria.** *Trop Med Int Health* 2001, **6:**92-98.

54. Njama-Meya D, Kamya MR, Dorsey G: **Asymptomatic parasitaemia as a risk factor for symptomatic malaria in a cohort of Ugandan children.** *Trop Med Int Health* 2004, **9:**862-8.

55. Paul RE, Hackford I, Brockman A, Muller-Graf C, Price R, Luxemburger C, White NJ, Nosten F, Day KP: **Transmission intensity and *Plasmodium falciparum* diversity on the northwestern border of Thailand.** *Am J Trop Med Hyg* 1998, **58:**195-203.

56. Bloland PB, Boriga DA, Ruebush TK, McCormick JB, Roberts JM, Oloo AJ, Hawley W, Lal A, Nahlen B, Campbell CC: **Longitudinal cohort study of the epidemiology of malaria infections in an area of intense malaria transmission II. Descriptive epidemiology of malaria infection and disease among children.** *Am J Trop Med Hyg* 1999, **60:**641-648.

57. Roper C, Elhassan IM, Hviid L, Giha H, Richardson W, Babiker H, Satti GM, Theander TG, Arnot DE: **Detection of very low level *Plasmodium falciparum* infections using the nested polymerase chain reaction and a reassessment of the epidemiology of unstable malaria in Sudan.** *Am J Trop Med Hyg* 1996, **54:**325-331.

58. Collins WE, Jeffery GM: **A retrospective examination of the patterns of recrudescence in patients infected with *Plasmodium falciparum*.** *Am J Trop Med Hyg* 1999, **61:**44-48.

59. Molineaux L, Gramiccia G: **Parasitology.** In *The Garki Project*. Geneva: World Health Organisation; 1980:109-172.

60. Anderson RM, May RM: **Indirectly transmitted microparasites.** In *Infectious Diseases of Humans:Dynamics and control*. Oxford: Oxford University Press; 1991:374-432.

61. Franks S, Koram KA, Wagner GE, Tetteh K, McGuinness D, Wheeler JG, Nkrumah F, Ranford-Cartwright L, Riley EM: **Frequent and persistent, asymptomatic *Plasmodium falciparum* infections in African infants, characterized by multilocus genotyping.** *J Infect Dis* 2001, **183:**796-804.

62. Bruce MC, Donnelly CA, Packer M, Lagog M, Gibson N, Narara A, Walliker D, Alpers MP, Day KP: **Age- and species-specific duration of infection in asymptomatic malaria infections in Papua New Guinea.** *Parasitology* 2000, **121 ( Pt 3):**247-256.

63. Babiker HA, Abdel-Muhsin AM, Ranford-Cartwright LC, Satti G, Walliker D: **Characteristics of *Plasmodium falciparum* parasites that survive the lengthy dry season in eastern Sudan where malaria transmission is markedly seasonal.** *Am J Trop Med Hyg* 1998, **59:**582-590.

64. Earle WC, Perez M, Del Rio J, Arzola C: **Observations on the course of naturally acquired malaria in Puerto Rico.** *Puerto Rico J Public Health Trop Med* 1939, **14:**391-406.

65. Sama W, Owusu-Agyei S, Felger I, Vounatsou P, Smith T: **An immigration-death model to estimate the duration of malaria infection when detectability of the parasite is imperfect.** *Stat Med* 2005, **24:**3269-3288.

66. White NJ: **Assessment of the pharmacodynamic properties of antimalarial drugs *In vivo*.** *Antimicrob Agents Chemother* 1997, **41:**1413-1422.

67. Day NP, Pham TD, Phan TL, Dinh XS, Pham PL, Ly VC, Tran TH, Nguyen TH, Bethell DB, Nguyan HP, Tran TH, White NJ: **Clearance kinetics of parasites and pigment-containing leukocytes in severe malaria.** *Blood* 1996, **88:**4694-4700.

68. Watt G, Shanks GD, Phintuyothin P: **Prognostic significance of rises in parasitaemia during treatment of falciparum malaria.** *Trans R Soc Trop Med Hyg* 1992, **86:**359-360.

69. Fontanet AL, Walker AM: **Predictors of treatment failure in multiple drug-resistant falciparum malaria: results from a 42-day follow-up of 224 patients in eastern Thailand.** *Am J Trop Med Hyg* 1993, **49:**465-472.

70. Nosten F, Luxemburger C, ter Kuile FO, Woodrow C, Eh JP, Chongsuphajaisiddhi T, White NJ: **Treatment of multidrug-resistant *Plasmodium falciparum* malaria with 3-day artesunate-mefloquine combination.** *J Infect Dis* 1994, **170:**971-977.

71. Maitland K, Williams TN, Bennett S, Newbold CI, Peto TE, Viji J, Timothy R, Clegg JB, Weatherall DJ, Bowden DK: **The interaction between *Plasmodium falciparum* and P. vivax in children on Espiritu Santo island, Vanuatu.** *Trans R Soc Trop Med Hyg* 1996, **90:**614-620.

72. Cox J: *Remote sensing pilot project, EC-CMCP Malaria Control Project in Cambodia.* 2002.

73. Sagara I, Sangare D, Dolo G, Guindo A, Sissoko M, Sogoba M, Niambele MB, Yalcoue D, Kaslow DC, Dicko A, Klion AD, Diallo D, Miller LH, Toure Y, Doumbo O: **A high malaria reinfection rate in children and young adults living under a low entomological inoculation rate in a periurban area of Bamako, Mali.** *Am J Trop Med Hyg* 2002, **66:**310-313.

74. Owusu-Agyei S, Koram KA, Baird JK, Utz GC, Binka FN, Nkrumah FK, Fryauff DJ, Hoffman SL: **Incidence of symptomatic and asymptomatic *Plasmodium falciparum* infection following curative therapy in adult residents of northern Ghana.** *Am J Trop Med Hyg* 2001, **65:**197-203.

75. McCombie SC: **Treatment seeking for malaria: a review of recent research.** *Soc Sci Med* 1996, **43:**933-945.

76. Munguti KJ: **Community perceptions and treatment seeking for malaria in Baringo district, Kenya: implications for disease control.** *East Afr Med J* 1998, **75:**687-691.

77. Nuwaha F: **People's perception of malaria in Mbarara, Uganda.** *Trop Med Int Health* 2002, **7:**462-470.

78. Deressa W, Ali A, Enqusellassie F: **Self-treatment of malaria in rural communities, Butajira, southern Ethiopia.** *Bull World Health Organ* 2003, **81:**261-268.

79. Espino F, Manderson L: **Treatment seeking for malaria in Morong, Bataan, the Philippines.** *Soc Sci Med* 2000, **50:**1309-16.

80. Yeung S, Van Damme W, Socheat D, White NJ, Mills A: **Access to Artemisinin combination therapy for malaria in remote areas of Cambodia.** *Malar J* 2007.

81. Ruebush TK, Kern MK, Campbell CC, Oloo AJ: **Self-treatment of malaria in a rural area of western Kenya.** *Bull World Health Organ* 1995, **73:**229-236.

82. Luxemburger C, Thwai KL, White NJ, Webster HK, Kyle DE, Maelankirri L, Chongsuphajaisiddhi T, Nosten F: **The epidemiology of malaria in a Karen population on the western border of Thailand.** *Trans R Soc Trop Med Hyg* 1996, **90:**105-111.

83. Drakeley CJ, Jawara M, Targett GA, Walraven G, Obisike U, Coleman R, Pinder M, Sutherland CJ: **Addition of artesunate to chloroquine for treatment of *Plasmodium falciparum* malaria in Gambian children causes a significant but short-lived reduction in infectiousness for mosquitoes.** *Trop Med Int Health* 2004, **9:**53-61.

84. Handunnetti SM, Gunewardena DM, Pathirana PP, Ekanayake K, Weerasinghe S, Mendis KN: **Features of recrudescent chloroquine-resistant *Plasmodium falciparum* infections confer a survival advantage on parasites and have implications for disease control.** *Trans R Soc Trop Med Hyg* 1996, **90:**563-567.

85. Robert V, Awono-Ambene HP, Le Hesran JY, Trape JF: **Gametocytemia and infectivity to mosquitoes of patients with uncomplicated *Plasmodium falciparum* malaria attacks treated with chloroquine or sulfadoxine plus pyrimethamine.** *Am J Trop Med Hyg* 2000, **62:**210-216.

86. Price RN, Nosten F, Luxemburger C, ter Kuile FO, Paiphun L, Chongsuphajaisiddhi T, White NJ: **Effects of artemisinin derivatives on malaria transmissibility.** *Lancet* 1996, **347:**1654-1658.

87. Adjuik M, Babiker A, Garner P, Olliaro P, Taylor W, White NJ: **Artesunate combinations for treatment of malaria: meta-analysis.** *Lancet* 2004, **363:**9-17.

88. Stepniewska K, Taylor WR, Mayxay M, Price R, Smithuis F, Guthmann JP, Barnes K, Myint HY, Adjuik M, Olliaro P, Pukrittayakamee S, Looareesuwan S, Hien TT, Farrar J, Nosten F, Day NP, White NJ: ***In vivo* Assessment of Drug Efficacy against *Plasmodium falciparum* Malaria: Duration of Follow-Up.** *Antimicrob Agents Chemother* 2004, **48:**4271-4280.

89. Collins WE, Jeffery GM: **A retrospective examination of the patterns of recrudescence in patients infected with *Plasmodium falciparum*.** *Am J Trop Med Hyg* 1999, **61:**44-48.

90. Rosenberg R, Andre RG, Ketrangsee S: **Seasonal fluctuation of *Plasmodium falciparum* gametocytaemia.** *Trans R Soc Trop Med Hyg* 1990, **84:**29-33.

91. Ijumba JN, Mosha FW, Lindsay SW: **Malaria transmission risk variations derived from different agricultural practices in an irrigated area of northern Tanzania.** *Med Vet Entomol* 2002, **16:**28-38.

92. Toma T, Miyagi I, Okazawa T, Kobayashi J, Saita S, Tuzuki A, Keomanila H, Nambanya S, Phompida S, Uza M, Takakura M: **Entomological surveys of malaria in Khammouane Province, Lao PDR, in 1999 and 2000.** *Southeast Asian J Trop Med Public Health* 2002, **33:**532-546.

93. Prakash A, Bhattacharyya DR, Mohapatra PK, Mahanta J: **Estimation of vectorial capacity of Anopheles dirus (Diptera: Culicidae) in a forest-fringed village of Assam (India).** *Vector Borne Zoonotic Dis* 2001, **1:**231-237.

94. Garrett-Jones C, Shidrawi GR: **Malaria vectorial capacity of a population of Anopheles gambiae: an exercise in epidemiological entomology.** *Bull World Health Organ* 1969, **40:**531-545.

95. Hay SI, Rogers DJ, Toomer JF, Snow RW: **Annual *Plasmodium falciparum* entomological inoculation rates (EIR) across Africa: literature survey, Internet access and review.** *Trans R Soc Trop Med Hyg* 2000, **94:**113-127.

96. Jeffery GM, Eyles DE: **Infectivity to mosquitoes of *Plasmodium falciparum* as related to gametocyte density and duration of infection.** *Am J Trop Med Hyg* 1955, **4:**781-789.

97. Drakeley CJ, Secka I, Correa S, Greenwood BM, Targett GA: **Host haematological factors influencing the transmission of *Plasmodium falciparum* gametocytes to Anopheles gambiae s.s. mosquitoes.** *Trop Med Int Health* 1999, **4:**131-138.

98. Bonnet S, Gouagna C, Safeukui I, Meunier JY, Boudin C: **Comparison of artificial membrane feeding with direct skin feeding to estimate infectiousness of *Plasmodium falciparum* gametocyte carriers to mosquitoes.** *Trans R Soc Trop Med Hyg* 2000, **94:**103-106.

99. Graves PM, Burkot TR, Carter R, Cattani JA, Lagog M, Parker J, Brabin BJ, Gibson FD, Bradley DJ, Alpers MP: **Measurement of malarial infectivity of human populations to mosquitoes in the Madang area, Papua, New Guinea.** *Parasitology* 1988, **96 ( Pt 2):**251-263.

100. Drakeley CJ, Akim NI, Sauerwein RW, Greenwood BM, Targett GA: **Estimates of the infectious reservoir of *Plasmodium falciparum* malaria in The Gambia and in Tanzania.** *Trans R Soc Trop Med Hyg* 2000, **94:**472-476.
