# Supplementary material for: Spread of anti-malarial drug resistance: Mathematical model with implications for ACT drug policies
Source: Malar J. 2008 Nov 2;7:229. doi: 10.1186/1475-2875-7-229 (PMC2585590; doi:10.1186/1475-2875-7-229)
Supplement: Additional file 3 — Sensitivity analysis results. [file 1475-2875-7-229-S3.doc]

**Supporting Information: Additional File 3 (Sensitivity analysis results)**

**Spread of anti-malarial drug resistance: Mathematical model with implications for ACT drug policies**

Authors: Wirichada Pongtavornpinyo, Shunmay Yeung, Ian M Hastings, Arjen M Dondorp, Nicholas PJ Day, Nicholas J White

**Table S9: The results of the sensitivity analysis shown as the descriptive statistics (mean, median, variance (var), standard deviation (SD), standard error (SE) and coefficient of variation (CV)) of malaria prevalence and resistance at year 2, 5, 8 and 10 in the four baseline scenarios (A – D).**

| **Output** | **Descriptive**  **statistics** | **Scenario** | | | |
| --- | --- | --- | --- | --- | --- |
|  |  | **A** | **B** | **C** | **D** |
| Prevalence_Y2_  (%) | Mean | 0.74  0.63  0.13  0.36  0.01  48.79 | 0.72  0.62  0.12  0.35  0.00  48.10 | 36.33  34.55  116.42  10.79  0.15  29.70 | 36.65  35.21  118.70  10.90  0.15  29.73 |
|  | Median |  |  |  |  |
|  | Var |  |  |  |  |
|  | SD |  |  |  |  |
|  | SE |  |  |  |  |
|  | CV |  |  |  |  |
| Prevalence_Y5_  (%) | Mean | 3.81  1.61  15.95  3.99  0.06  104.83 | 0.70  0.56  0.25  0.50  0.01  71.67 | 41.27  40.04  111.24  10.55  0.15  25.56 | 40.53  39.37  117.35  10.83  0.15  26.73 |
|  | Median |  |  |  |  |
|  | Var |  |  |  |  |
|  | SD |  |  |  |  |
|  | SE |  |  |  |  |
|  | CV |  |  |  |  |
| Prevalence_Y8_  (%) | Mean | 4.60  3.47  14.93  3.86  0.05  83.96 | 0.88  0.65  0.47  0.68  0.01  77.86 | 43.20  42.20  112.45  10.60  0.15  24.54 | 42.11  41.04  119.37  10.93  0.15  25.95 |
|  | Median |  |  |  |  |
|  | Var |  |  |  |  |
|  | SD |  |  |  |  |
|  | SE |  |  |  |  |
|  | CV |  |  |  |  |
| Prevalence_Y10_  (%) | Mean | 4.63  3.52  14.74  3.84  0.05  82.87 | 0.91  0.68  0.48  0.69  0.01  75.89 | 43.62  42.65  112.11  10.59  0.15  24.27 | 42.44  41.48  119.83  10.95  0.15  25.79 |
|  | Median |  |  |  |  |
|  | Var |  |  |  |  |
|  | SD |  |  |  |  |
|  | SE |  |  |  |  |
|  | CV |  |  |  |  |
| Resistance_Y2_  (%) | Mean | 3.21  2.20  8.21  2.87  0.04  89.29 | 3.04  2.12  5.96  2.44  0.03  80.26 | 2.46  1.83  3.15  1.77  0.03  72.14 | 2.40  1.77  3.01  1.74  0.02  72.21 |
|  | Median |  |  |  |  |
|  | Var |  |  |  |  |
|  | SD |  |  |  |  |
|  | SE |  |  |  |  |
|  | CV |  |  |  |  |
| Resistance_Y5_  (%) | Mean | 71.55  86.85  952.18  30.86  0.44  43.13 | 33.99  24.64  587.88  24.25  0.34  71.34 | 46.23  39.70  1454.48  38.14  0.54  82.50 | 43.87  33.80  1404.80  37.48  0.53  85.44 |
|  | Median |  |  |  |  |
|  | Var |  |  |  |  |
|  | SD |  |  |  |  |
|  | SE |  |  |  |  |
|  | CV |  |  |  |  |
| Resistance_Y8_  (%) | Mean | 96.49  99.93  74.91  8.65  0.12  8.97 | 65.25  67.95  795.96  28.21  0.40  43.24 | 69.43  94.93  1456.21  38.16  0.54  54.96 | 67.12  91.73  1492.60  38.63  0.55  57.56 |
|  | Median |  |  |  |  |
|  | Var |  |  |  |  |
|  | SD |  |  |  |  |
|  | SE |  |  |  |  |
|  | CV |  |  |  |  |
| Resistance_Y10_  (%) | Mean | 99.30  99.98  7.56  2.75  0.04  2.77 | 78.25  88.97  578.19  24.05  0.34  30.73 | 77.01  99.41  1231.03  35.09  0.50  45.56 | 74.80  98.79  1302.96  36.10  0.51  48.26 |
|  | Median |  |  |  |  |
|  | Var |  |  |  |  |
|  | SD |  |  |  |  |
|  | SE |  |  |  |  |
|  | CV |  |  |  |  |

**Table S10: Three parameters with the highest PRCCs to the outcomes from the model in all four baseline scenarios (A – D). For each model outputs, the three parameters with the highest PRCCs that have the absolute value greater or equal 0.2 are shown. The values of PRCCs are given in the brackets. The parameter codes are given in Table S1 – S6.**

| **Output** | **Scenario** | | | |
| --- | --- | --- | --- | --- |
|  | **A** | **B** | **C** | **D** |
| prevalence_Y2_  (%) | V1 (0.6)  IM4,S4,IM10 (0.2)  S1,M9 (-0.2) | IM4,S4,IM10 (0.2)  S1,M9 (-0.2) | A2 (0.4)  IM4,A1,S4,IM10 (0.3)  M9 (-0.3) | A2 (0.4)  IM4,A1,S4,IM10 (0.3)  M9 (-0.3) |
| prevalence_Y5_  (%) | V1 (0.7)  IM4,S4,M9 (0.2) | V1 (0.6)  IM4,S4,IM10 (0.2)  S1 (-0.2) | A1,A2 (0.4)  IM4,S4,IM10 (0.3)  V1 (0.2) | A1,A2 (0.4)  IM4,S4,IM10 (0.3) |
| prevalence_Y8_  (%) | V1 (0.7)  IM4,I7,S4 (0.2) | V1 (0.6)  IM4,S4,IM10 (0.2)  S2 (-0.2) | A1,A2 (0.4)  IM4,S4,IM10 (0.3)  V1 (0.2) | A1,A2 (0.4)  IM4,S4,IM10 (0.3) |
| prevalence_Y10_  (%) | V1 (0.7)  IM4,A1,I7,S4 (0.2) | V1 (0.6)  IM4,S4 (0.2)  S2 (-0.2) | A1,A2 (0.4)  IM4,S4,IM10 (0.3)  V1 (0.2) | A1,A2 (0.4)  IM4,S4,IM10 (0.3)  V1 (0.2) |
| resistance_Y2_  (%) | V1 (0.7)  IM4,S4,M9 (0.2) | V1 (0.7)  IM4,S4,M9 (0.2) | M9 (0.9) | M9 (0.9) |
| resistance_Y5_  (%) | V1 (0.7)  S1,S4,M9 (0.2) | V1 (0.6)  S4,IM8,M9 (0.2)  S2,IM10 (-0.2) | M9 (0.9)  IM10 (-0.2) | M9 (0.9)  IM10 (-0.2) |
| resistance_Y8_  (%) | S1 (0.4)  IM10 (-0.2) | V1 (0.6)  M9 (0.3)  S4,IM8 (0.2)  S2,IM10 (-0.2) | M9 (0.9)  IM10 (-0.2) | M9 (0.9)  IM10 (-0.2) |
| resistance_Y10_  (%) | V1 (0.3) | V1 (0.6)  M9 (0.3)  S4,IM8 (0.2)  S2,IM10 (-0.2) | M9 (0.8)  IM10 (-0.2) | M9 (0.9)  IM10 (-0.2) |
